# Comparison of Rheological, Microstructural, and Textural Properties of Set‐Type Yogurts Fermented With Novel *Streptococcus thermophilus* Strains From Yogurts Produced Mountain Villages of Turkey With Those Produced With Commercial Cultures

**DOI:** 10.1002/fsn3.70269

**Published:** 2025-06-12

**Authors:** Begüm Denktaş, Hale İnci Öztürk, Talha Demirci, Meryem Kübra Satılmış, Nihat Akın

**Affiliations:** ^1^ Department of Food Technology Vocational School, İstanbul Esenyurt University İstanbul Türkiye; ^2^ Department of Food Engineering Yıldız Technical University Istanbul Türkiye; ^3^ Department of Food Engineering Selcuk University Konya Türkiye; ^4^ Çine Vocational School Aydın Adnan Menderes University Aydın Türkiye

**Keywords:** LAB, rheology, *S. thermophilus*, texture, yogurt

## Abstract

In recent years, many consumers prefer artisanal cultures as they provide special characteristic taste and flavor compared to commercial ones. Therefore, it is important to preserve traditional cultures and use them in production to reduce dependence on commercial cultures and to develop consumer‐pleasing products. This study aimed to evaluate the technological performance of *Streptococcus thermophilus* strains isolated from yogurts collected from villages in Konya province, and to assess their suitability for commercial yogurt production. Physicochemical, microbiological, textural, rheological, microstructural, and sensory properties of experimental samples were analyzed for 28 days. During the refrigerated time at 4°C*, S* *. thermophilus* counts varied between 8.41 and 9.94 log CFU/g. The impact of different *S. thermophilus* isolates on textural properties was evident, with yogurt samples C, D, and B exhibiting the best performance across all textural parameters. The same situation was observed for microstructure; C, D, and E had the best microstructure images, supported by high water retention capacity and low syneresis degree. B yogurt received the highest general acceptability scores across all storage periods. The results suggested that the strains 10K1, 10K2, and 4K6 could be recommended as potential starter cultures for commercial yogurt production. Novel cultures do not always achieve the flavor and product‐specific quality characteristics that consumers like. However, it is very promising that some *S. thermophilus* strains isolated in this study show better properties than commercial cultures.

## Graphical abstract

Yogurt trials were carried out with novel *Streptococcus thermophilus* cultures. Some yogurt samples received higher acceptability than control yogurt. These new *S. thermophilus* cultures were found suitable for commercial production.

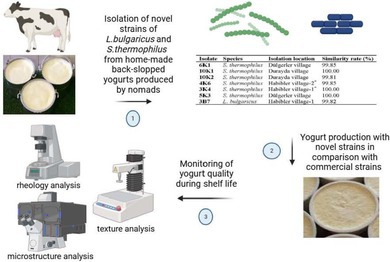

## 1 Introduction

Nowadays, at industrial level, starter cultures are preferred to ensure the reproducibility of fermented products (Sionek et al. 2023). Starter cultures are defined as preparations consisting of one or more specific species of microorganisms with high cell density, used in raw or pasteurized products to produce fermented foods by accelerating and directing the fermentation process through their metabolic or enzymatic activities (Fox et al. 2017; Bezie and Regasa 2019; García‐Díez and Saraiva 2021). Microorganisms used as starter culture are bacteria, molds and yeasts.

An important fermentation process in which starter cultures are mainly used is yogurt production. According to the Turkish Food Codex (TFC) Communiqué on Fermented Dairy Products, yogurt is a fermented milk product in which *S. thermophilus* and *L. bulgaricus* are used together as specific starter cultures in fermentation, which is obtained in unbroken (set) or stirred (stirred) form which contains a sufficient number of live and active starter bacteria at the last consumption date (Communiqué 2022). Starter cultures used in yogurt production must be free from health risks and should demonstrate optimal acid production, balanced proteolytic activity, and preferably resistance to bacteriophages substances (Celik et al. 2021). Additionally, they should be capable of producing flavor compounds, exopolysaccharides (EPSs), and antimicrobial substances (İspirli and Dertli 2018). Starter cultures can offer a number of advantages compared to spontaneous yogurt fermentation, including maintaining product quality and consistency, controlling fermentation, increasing fermentation yields, and reducing the risk of the growth of pathogenic microorganisms (Ayivi and Ibrahim 2022; Sionek et al. 2023). To ensure efficient fortification of fermented dairy products, bacterial strains should be carefully selected as they have many benefits but may also have some disadvantages. For example, some strains commonly found in fermented foods may be folate consumers, thus reducing the bioavailability of the final product (González‐González et al. 2022).

Today, both spontaneous and controlled fermentations with the participation of microorganisms naturally present in raw materials are used to obtain safer products; spontaneous fermentation products are a suitable source for the isolation of new strains with functional properties (González‐González et al. 2022; Sionek et al. 2023). There has been a trend towards the selection and development of new cultures from autochthonous microbiota of spontaneously fermented food products for use in the industrial production (Andreson et al. 2024). Consumers generally prefer natural organisms and foods over genetically modified ones. In the light of this information, LAB are directly isolated from various natural foods and traditional fermented foods, such as yogurt, to develop starter and/or probiotic cultures that can enhance the sensory properties of the products they are applied to (Hajimohammadi Farimani et al. 2016; Celik et al. 2021).

Considering these objectives, the aim of this study is to protect the local yogurt culture heritage by ensuring the production of products with preserved traditional characteristics and to create a response and alternative to the increasing interest in traditional products by isolating LAB from village‐type yogurts and manufacturing products with these cultures. The present study investigated previously isolated and characterized *S. thermophilus* isolates to assess their potential for commercial application. The cultures used in this study had previously been evaluated in commercial‐scale yogurt production trials; however, due to the structural differences observed in the resulting products, this study was grounded in the aim of elucidating the effects of combining different *S. thermophilus* strains with the most suitable *L. bulgaricus* strain on yogurt structure. For this purpose, the textural, microstructural, rheological, microbiological, and physicochemical properties of yogurts fermented with different *S. thermophilus* strains over a 28‐day cold storage period at 4°C were analyzed, focusing on the impact of culture variation on these parameters. Additionally, the sensory evaluation of the yogurts was also evaluated.

## 2 Materials and Methods

### 2.1 Materials

In this study, previously isolated yogurt cultures, consisting of 6 *S. thermophilus* and 1 *L. bulgaricus* strain, were used. All these LAB were isolated from artisanal back‐slopped yogurts produced in Habiller, Durayda, and Dülgerler villages of Konya (Türkiye). The reason why yogurt samples were collected from the mountainous regions of Konya is that this region is difficult to reach and contact with the city is not frequent. If the samples were not selected in this way, these yogurts would be contaminated with commercial cultures or produced using commercial cultures. By planning in this way, the mixing of commercial cultures to artisanal yogurts was prevented. The villagers produced the yogurts with the back‐slopping method before 1–2 days of collection. The yogurt milk used in yogurt production (pasteurized and evaporated, 14% total dry matter, pH 6.60) was provided from Panagro Meat and Milk Integrated Facilities.

### 2.2 Culture Isolation and Identification

**FIGURE 1:**
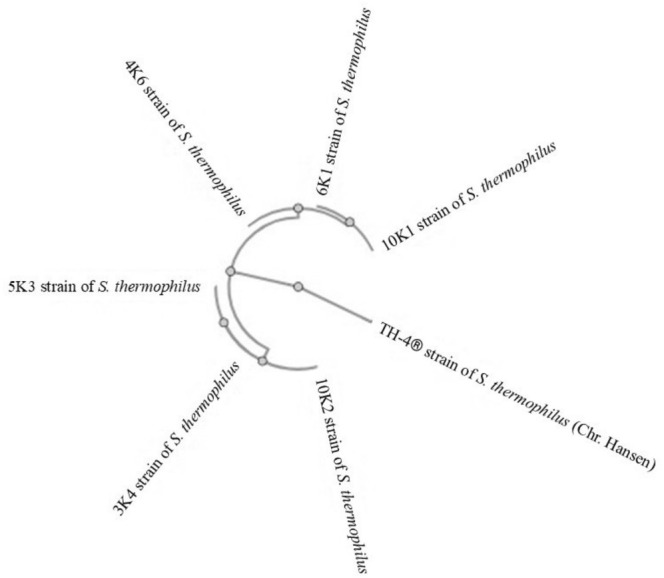
Phylogenetic relationships among *Streptococcus thermophilus* isolates used in this study.

These strains were isolated using the spread‐plate and streaking methods on both MRS and M17 medium (Merck). The isolated cultures were checked regularly for purity using a microscope and were identified by 16S rRNA sequencing. In this context, F365 (5′‐ACWCCTACGGGWGGCWGC‐3′) and R1064 (5′‐AYCTCACGRCACGAGCTGAC‐3′) universal primers, designed from an invariant region in the 16S rRNA sequences for LAB, were obtained from the Sentebiolab, Turkey. The PCR amplification was performed in a final 30 μL reaction volume using a BioRAD thermal cycler (T100, Foster City, CA, USA). The PCR conditions for the amplification procedure were as follows: initial denaturation at 95°C for 5 min, 35 cycles of denaturation at 95°C for 30 s, primer annealing at 58°C for 30 s, and extension at 72°C for 45 s, and one cycle of final extension at 72°C for 10 min. The presence of PCR products and their purity were verified by agarose gel electrophoresis (Özkan et al. 2021). Isolates were identified by comparing the sequence results with the DNA sequence database present at the National Centre for Biotechnology Information (NCBI) using the BLAST algorithm. The information regarding the bacteria used in production is presented in Table 1 and their phylogenetic tree is illustrated in Figure 1.

**TABLE 1:** Characteristics of bacteria used in yogurt production and yogurt sample codes.

Yogurt code	Isolates *S. thermophilus*	Isolation location	Similarity rate (%)	Isolate *L. bulgaricus*	Isolation location	Similarity rate (%)
A	6K1	Dülgerler village	99.85	3B7	Habibler village‐1^a^	99.82
B	10K1	Durayda village	100.00	3B7	Habibler village‐1^a^	99.82
C	10K2	Durayda village	99.81	3B7	Habibler village‐1^a^	99.82
D	4K6	Habibler village‐2^a^	99.85	3B7	Habibler village‐1^a^	99.82
E	3K4	Habibler village‐1^a^	100.00	3B7	Habibler village‐1^a^	99.82
F	5K3	Dülgerler village	100.00	3B7	Habibler village‐1^a^	99.82
Control	Commercial culture	—	—	Commercial culture	—	—

^a^ Expressions 1 and 2 used for the same regions represent yogurts from different producers.

### 2.3 Yogurt Production

Cultures activated the day before were placed separately in 2% TW60 media (6%, sterilized at 95°C for 30 min) and incubated until the pH reached 4.6. Following this process, 2% was taken from the incubated TW60 medium, added to the new sterile TW60 medium, and incubated until the pH reached 4.60. Yogurt milk was heated to 43°C, and 2% yogurt starter cultures from the prepared TW60 cultures were added to the yogurt milk (1:1, *S. thermophilus* : *L. bulgaricus* ) and incubated at 43°C until the pH reached 4.60. Following incubation, the yogurts were cooled for 30 min and stored under refrigerator conditions for a shelf‐life of 28 days (Sözeri Atik et al. 2023). All yogurts were produced in 100 mL sterile sample containers. 40 L of yogurt milk was used, and a total of 380 yogurt units of 100 mL each were produced.

In experimental design, six different *S. thermophilus* strains and one *L. bulgaricus* strain were used in yogurt production. Accordingly, a total of seven yogurt samples were produced, including the control group. The experimental codes and bacterial combinations of yogurt samples are shown in Table 1. Analyses during shelf‐life were made on the 1st, 7th, 14th, and 28th days. The pH, titratable acidity (TA), water‐holding capacity, syneresis, textural analyses, rheological analyses, and microbiological analyses were conducted periodically, while dry matter, ash, protein, fat content, and microstructural analyses were performed on the 28th day of storage, and sensory analysis was carried out on the 14th day of storage.

### 2.4 pH And TA Analysis

Before measuring the pH of the yogurt samples, the pH meter (WTW 315i Set brand) was calibrated. After calibration, measurement was carried out by dipping the pH meter probe into the sample yogurt. For TA values of yogurt samples, 10 mL of yogurt sample was diluted 1:1 with distilled water and titrated with 0.1 N NaOH until the pH meter showed 8.20. TA was determined as % lactic acid content (Yang and Yoon 2022).

### 2.5 Water Holding Capacity and Syneresis

Water holding capacity of yogurt samples was determined using the method specified by Ranadheera et al. (2012). For this purpose, 5 g of yogurt sample was centrifuged for 30 min at 4500 rpm at +10°C, and the amount of separated serum was determined by weighing. The results were reported as a percentage. Syneresis values of yogurts were used by modifying the method specified by Isanga and Zhang (2009). For this purpose, 20 g of yogurt sample was left to filter through filter paper (Whatman no:1, International Ltd., Maidstone, England) placed in glass funnels for 4 h at +4°C. At the end of this period, the separated serum was weighed and calculated as apercentage.

### 2.6 Chemical Composition Analysis

For the total dry matter amount, it was determined gravimetrically. The amount of fat in yogurts was determined using the Gerber method according to the AOAC (2005). To determine the ash content of yogurt samples, approximately 3 g of yogurt sample was weighed into the porcelain crucible. Then, the samples were burned in a muffle furnace at 550°C. Following this application, the crucibles were cooled in a desiccator and weighed to determine the % ash rate (Atallah et al. 2020). Total nitrogen amount was determined by the Kjeldahl method. Protein value was determined by multiplying the amount of nitrogen by the coefficient of 6.38 (Atallah et al. 2020).

### 2.7 Microbiological Analysis

To evaluate the viability of yogurt cultures throughout their shelf‐life, the total number of live *L. bulgaricus* and *S. thermophilus* strains was determined. *L. bulgaricus* and *S. thermophilus* enumeration was carried out by the spread plate method (Tharmaraj and Shah 2003). To determine the number of *L. bulgaricus* , serial dilutions prepared from yogurts were spread onto MRS agar (pH 5.4, Merck KGaA, Darmstadt, Germany) and incubated under anaerobic conditions at 43°C for 48 h. For *S. thermophilus* , M17 agar (7.20 ± 0.20, Merck KGaA, Darmstadt, Germany) was used and the inoculated petri dishes were incubated under aerobic conditions at 43°C for 48 h. Following incubation, all colonies were counted and the total number of live *L. bulgaricus* and *S. thermophilus* was given in log CFU/g. At the same time, Potato Dextrose Agar (PDA) (Merck) was used to count yeasts and petri dishes were placed at 25°C for 3–5 days.

### 2.8 Texture Profile Analysis

Measurements of yogurt samples were made using the TX.2TA Texture Profile Analyzer (Stable Micro Systems, Godalming, UK) device and back extrusion unit. With this analysis, hardness, consistency, internal stickiness, and viscosity index values of yogurt samples were measured. The device was operated with a 5 kg load cell, and the samples were subjected to penetration testing under a 20 mm cylindrical probe directly in a 100 mL sample container. The sinking depth was applied as 10 mm, and the sinking speed was 10 mm/s. Analysis values were recorded as firmness (g), consistency (g.s), cohesiveness (g), and viscosity (g.s).

### 2.9 Rheological Analysis

Rheological properties of yogurt samples were evaluated using a stress‐controlled rotational rheometer (Malvern Instruments Ltd., Kinexus Proþ, Worcestershire, UK) with a parallel plate (20 mm diameter, PU20 SR2453 SS). For this analysis, yogurt samples were carefully taken from the container without damaging the structure of the set‐type yogurts. The gap was set to 2 mm and measurements were made at 4°C. Frequency sweep tests were performed at 0.01–10 Hz and 2% strain to determine storage (*G*′), loss modulus (*G*″), complex viscosity (μ*) and complex modulus (G*). Experiments were performed within the linear viscoelastic (LVE) region to prevent degradation of the gel structure (Sözeri Atik et al. 2023).

### 2.10 Microstructure Analysis

Microstructure analysis was carried out by modifying the method specified by Pang et al. (2014). For this purpose, 0.1% Rhodamine B (M = 479.02 g/mol, CAS‐No: 81‐88‐9, Merck) fluorescent dye (each 1 mL sample) was added to the milk to be processed into yogurt before starting fermentation to dye the protein network in the milk. After being distributed homogeneously, it was incubated at 43°C. After incubation, it was stored under refrigerator conditions (+4°C) for 28 days. A piece of yogurt was taken without damaging the structure and transferred to the slide. The slide was covered with a coverslip. Then the protein network was imaged under a laser scanning confocal microscope (Nikon A1, Software: NIS elements AR 4.20.03 64‐bit).

### 2.11 Sensory Analysis

The sensory evaluation was conducted by modifying the method of Oraç and Akın (2019). The panel consisted of 11 experienced academicians (from the Food Engineering Department at Selçuk University, 6 women and 5 men, age range 25–40), all of whom have expertise in sensory analysis. Yogurt samples were coded and presented to the panelists in random order. Sensory analyses were performed in the same environment (same room temperature, humidity and light conditions were constant). Yogurts were presented to each panelist in different coding and order as known only to the analyst. The panelists evaluated the samples, scoring them on a 9‐point hedonic scale (1—dislike extremely/unacceptable, 2—dislike very much, 3—dislike, 4—dislike slightly, 5—neither like nor dislike, 6—like slightly, 7—like, 8—like very much and 9—like extremely/excellent) based on attributes such as color, appearance, odor, texture, taste, and overall acceptability. The panelists were given quality criteria such as non‐uniform color, free whey and unnatural color as quality criteria for color and appearance to use in their sensory evaluation, while criteria such as cooked flavor, creamy, fermented, salty, and foreign were used for odor and taste. The panelists also used criteria such as too thin, drinkable, granular, ropy, and too firm to evaluate the texture.

### 2.12 Statistical Analysis

All data are given as the arithmetic mean and SD of two replicates and two parallels. The results were subjected to one‐way ANOVA in the statistical program Minitab 16 (State College, USA) to determine significant differences between samples and storages. Significant differences between results were compared with the Tukey test at the 95% confidence interval.

## 3 Results

### 3.1 pH And TA of Yogurt Samples

**FIGURE 2:**
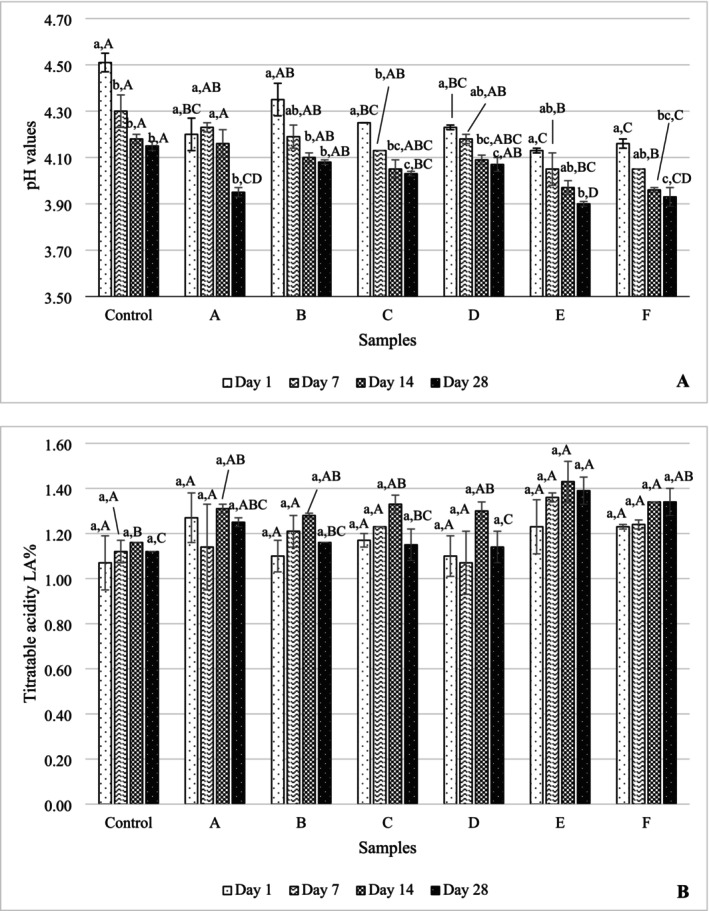
The pH (A) and titratable acidity (B) values of 28 days of cold storage of yogurt samples. The values given are the arithmetic mean of 2 replicates and 2 parallels and express the arithmetic mean ± standard deviation. Significant differences were determined by the Tukey test at the *p* ≤ 0.05 level. Comparisons between storages are indicated in lowercase, and comparisons between samples are expressed in capital letters. Sample codes refer to A: 6K1‐3B7, B: 10K1‐3B7, C: 10K2‐3B7, D: 4K6‐3B7, E: 3K4‐3B7, F: 5K3‐3B7, Control: Commercial culture combination.

pH values of yogurt samples were measured throughout their shelf life, and the values are given in Figure 2. On the first day of storage, pH varied between 4.13 and 4.51, and this difference was statistically insignificant (*p* > 0.05). A decrease in pH values was observed during the shelf life due to the continued development of yogurt bacteria throughout storage. TA results of yogurt samples throughout shelf life are given in Figure 2. TA values of yogurt samples varied between 1.07% and 1.43% lactic acid during the storage period. A significant difference was found in the comparison between samples in A, B, C, D, F, and K yogurt samples on the 14th and 28th days of storage (*p* ≤ 0.05).

### 3.2 WHC and Syneresis of Yogurt Samples

WHC of yogurt samples were analyzed throughout their shelf‐life and the results were given in Table 2. At the beginning of the storage period, the highest WHC was observed in B yogurt with a value of 55.50%, while at the end of the storage it was observed in F yogurt with a value of 55.90%. The difference between all samples during the storage period was found to be statistically significant (*p* ≤ 0.05). When the first day was compared to the 28th day, there was a statistically significant increase in the WHC of C and F samples (*p* ≤ 0.05).

**TABLE 2:** The syneresis (%) of yogurt samples during cold storage.

Samples^a^	Storage time			
	Day 1	Day 7	Day 14	Day 28
*Syneresis*				
Control	30.39 ± 2.10^A.a^	24.44 ± 1.82^A.ab^	18.89 ± 1.40^A.bc^	12.96 ± 0.79^A.c^
A	18.09 ± 1.96^C.ab^	24. 12 ± 1. 66^AB.a^	13.82 ± 1.66^bc.b^	13.25 ± 0.84^A.b^
B	21. 07 ± 0.81^bc.a^	18.84 ± 1.68^B.a^	11.27 ± 0.95^C.b^	12.56 ± 1.71^A.b^
C	25.75 ± 0.91^AB.a^	20.20 ± 1.48^AB.b^	15.09 ± 0.65^ABC.bc^	10. 82 ± 1. 80^A.c^
D	28.26 ± 1.93^A.a^	23. 19 ± 0.69^AB.a^	15.81 ± 1.15^ABC.b^	11.77 ± 1.37^A.b^
E	26.34 ± 0.48^AB.a^	21.93 ± 0.42^AB.b^	14.23 ± 1.48^ABC.c^	11.65 ± 0.91^A.c^
F	25.62 ± 0.53 ^AB. a^	22.66 ± 0.94^AB.a^	16. 37 ± 0.67^AB.b^	12.20 ± 1.09^A.c^

*Note:* Values are provided as mean ± SD. Significant differences (*p* ≤ 0.05) between yogurt samples were determined with the Tukey test. Comparisons between storage conditions are expressed in lower case; comparisons between samples are expressed in upper case.

^a^ Control: Commercial culture combination, A: 6K1‐3B7, B: 10K1‐3B7, C: 10K2‐3B7, D: 4K6‐3B7, E: 3K4‐3B7, F: 5K3‐3B7.

Syneresis tendency of yogurt samples was evaluated throughout their shelf‐life, and the results were given in Table 2. Since samples with high WHC showed less syneresis tendency, the WHC results were found to be compatible with the syneresis results. Whilst the lowest serum separation was observed in sample A (18.09%), the highest syneresis values were in control (30.39%) and sample D (28.26%) on the first day of storage. It was determined that serum separation decreased in almost all samples as storage time progressed. While the control yogurt sample had the highest syneresis levels until day 14, the syneresis values of all yogurts were statistically insignificantly different from each other on day 28 (*p* ≤ 0.05).

### 3.3 Chemical Composition Content of Yogurt Samples

Dry matter, fat, protein, and ash analysis of yogurt samples were performed only on the 28th day of storage and the results are given in Table 3. The dry matter content of yogurt samples produced novel LAB cultures was 14.39%–14.68%, the fat content was 4.76%–4.20%, the protein amount was 3.27%–3.68% and the ash content was 0.81%–1.00%. The differences in dry matter, fat, protein, and ash amounts between all yogurt samples were statistically insignificant (*p* > 0.05).

**TABLE 3:** Physicochemical properties of yogurt samples.

Samples^a^	Dry matter (%)	Fat (%)	Protein (%)	Ash (%)
Control	14.61 ± 0.19	4.20 ± 0.02	3.53 ± 0.01	0.81 ± 0.21
A	14.43 ± 0.10	4.52 ± 0.26	3.33 ± 0.12	0.83 ± 0.24
B	14.48 ± 0.14	4.76 ± 0.08	3.31 ± 0.03	1.16 ± 0.23
C	14.68 ± 0.09	4.24 ± 0.03	3.27 ± 0.11	0.82 ± 0.23
D	14.65 ± 0.02	4.31 ± 0.11	3.51 ± 0.07	0.98 ± 0.01
E	14.42 ± 0.28	4.21 ± 0.08	3.57 ± 0.29	0.81 ± 0.21
F	14.39 ± 0.02	4.35 ± 0.25	3.68 ± 0.09	1.13 ± 0.23

*Note:* Values are provided as mean ± SD. Statistical differences between yogurt samples were determined with the Tukey test. No significant differences were observed among the groups (*p* > 0.05).

^a^ Control: Commercial culture combination, A: 6K1‐3B7, B: 10K1‐3B7, C: 10K2‐3B7, D: 4K6‐3B7, E: 3K4‐3B7, F: 5K3‐3B7.

### 3.4 Microbiology Analysis of Yogurt Samples

**FIGURE 3:**
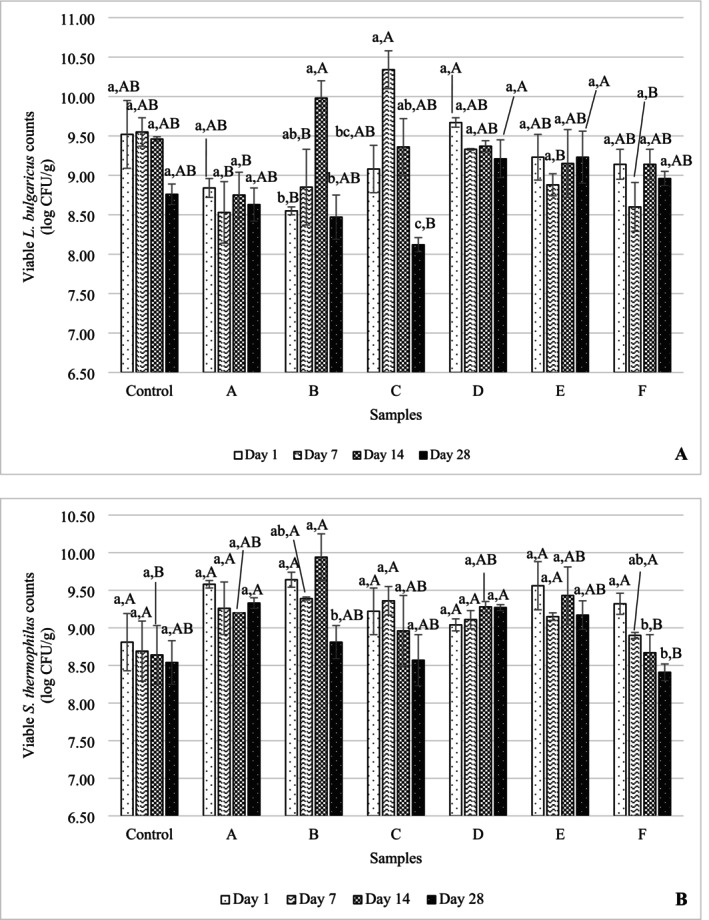
Microbial changes of yogurt samples according to (A) *Lactobacillus bulgaricus* and (B) *Streptococcus thermophilus* during 28 days of storage. The values given are the arithmetic mean of two replicates and two parallels and express the arithmetic mean ± SD. Significant differences were determined by the Tukey test at the *p ≤* 0.05 level. Comparisons between storages are indicated in lowercase and comparisons between samples are expressed in capital letters. Sample codes refer to A: 6K1‐3B7, B: 10K1‐3B7, C: 10K2‐3B7, D: 4K6‐3B7, E: 3K4‐3B7, F: 5K3‐3B7, Control: Commercial culture combination.

The counts of *L. bulgaricus* and *S. thermophilus* in the yogurt samples were monitored throughout the shelf‐life, and the enumeration of yeasts and molds was also carried out. No yeasts or molds growth was observed in the samples during shelf‐life. The number of *L. bulgaricus* determined in yogurt samples is presented in Figure 3A. On the first day of the storage, the highest number of *L. bulgaricus* was observed in sample D, followed by control, E, and F samples. Although a decrease in *L. bulgaricus* counts was detected in all yogurt samples from the beginning to the end of storage, the counts remained in the range of 8.12–10.34 log CFU/g throughout storage.

The number of *S. thermophilus* determined in yogurt samples throughout the storage period is presented in Figure 3B. During the storage, the number of *S. thermophilus* varied between 8.41 and 9.94 log CFU/g. When these measured values are compared with the number of live bacteria written in the TFC Fermented Dairy Products Communiqué, the number of *S. thermophilus* during the storage period of yogurts produced with novel cultures is among the desired values (> 7.00 log CFU/g). At the beginning of storage, the highest number of *S. thermophilus* was determined in samples B, A, and E (> 9.50 log CFU/g) and a statistically insignificant difference was observed between control yogurt and yogurt produced with indigenous cultures in terms of *S. thermophilus* count (*p* > 0.05). The samples containing the highest number of *S. thermophilus* on the 28th day of storage were determined as A, D, E, and B. In all samples except sample D, the number of streptococci was found to be lower at the end of storage compared to the beginning of storage. In sample D, it was observed that the number of streptococci increased at the end of storage.

### 3.5 Texture Profile Analysis of Yogurt Samples

TPA results of yogurt samples are shown in Table 4. Firmness values of yogurt samples at the beginning of storage were found to be between 674.3 and 1108.0 g. While the highest firmness values were determined in samples C, B, and E at the beginning of storage, they were highest in samples C, D, and B on the last day of storage. Considering all storage, the weakest yogurt sample in terms of firmness is the control sample. At the same time, when comparing all samples, the difference between the firmness values of yogurt samples on the 14th and 28th days during the storage period was statistically significant (*p* ≤ 0.05). At the beginning of the storage period, the highest consistency value was observed in sample C, followed by samples B and A. The samples with the lowest consistency values were observed in the control, F, and D samples where the firmness was also determined to be low. At the end of the 28‐day storage period, the highest consistency value was determined in sample C, followed by samples D, B, and E. On the first day of storage, the highest cohesiveness value was determined in the control group, followed by D yogurt samples, while the highest ones were in F, E, and control yogurts, respectively, at the end of the storage. However, these differences were found to be statistically insignificant.

**TABLE 4:** Textural characteristics of yogurt samples.

Samples^a^	Storage time (day)	Firmness (g)	Consistency (g.s)	Cohesiveness (g)	Viscosity (g.s)
Control	1	674.3 ± 142.8^A.a^	11,616 ± 2197^A.a^	111.68 ± 10.83^A.a^	64.61 ± 31.92^A.a^
	7	706.5 ± 104.6^A.a^	11,845 ± 1302^B.a^	105.91 ± 3.73^A.a^	137.12 ± 13.41^A.a^
	14	702.0 ± 51.1^B.a^	11,756 ± 652^B.a^	107.99 ± 4.09^A.a^	160.19 ± 31.05^A.a^
	28	801.3 ± 21.1^C.a^	13,003 ± 410^D.a^	108.38 ± 0.11^A.a^	102.82 ± 38.19^A.a^
A	1	869.6 ± 58.2^A.a^	14,572 ± 362^A.a^	99.12 ± 7.06^A.a^	119.69 ± 122.59^A.a^
	7	1108.0 ± 269.9^A.a^	14,606 ± 223^AB.a^	106.24 ± 2.85^A.a^	61.16 ± 43.55^A.a^
	14	908.0 ± 3.7^AB.a^	14,424 ± 150^AB.a^	109.12 ± 0.47^A.a^	98.20 ± 108.96^A.a^
	28	932.1 ± 10.1^AB.a^	14,875 ± 261^bc.a^	106.68 ± 5.47^A.a^	56.27 ± 59.78^A.a^
B	1	944.4 ± 92.7^A.a^	15,036 ± 1742^A.a^	107.63 ± 8.24^A.a^	127.13 ± 108.95^A.a^
	7	955.5 ± 126.8^A.a^	15,185 ± 1398^AB.a^	104.38 ± 5.54^A.a^	97.67 ± 127.55^A.a^
	14	925.4 ± 98.8^AB.a^	14,991 ± 1295^A.a^	104.60 ± 6.51^A.a^	172.51 ± 16.57^A.a^
	28	991.5 ± 15.3^A.a^	15,654 ± 249^AB.a^	100.73 ± 3.95^A.a^	177.20 ± 15.94^A.a^
C	1	975.3 ± 112.9^A.a^	15,734 ± 1483^A.a^	107.14 ± 6.17^A.a^	139.41 ± 49.67^A.a^
	7	998.8 ± 11.4^A.a^	15,787 ± 758^A.a^	103.84 ± 0.61^A.a^	125.65 ± 28.92^A.a^
	14	1016.7 ± 79.1^A.a^	16,000 ± 1054^A.a^	113.33 ± 1.24^A.a^	139.93 ± 42.20^A.a^
	28	1027.9 ± 15.1^A.a^	16,626 ± 22^A.a^	100.72 ± 1.23^A.a^	154.00 ± 61.82^A.a^
D	1	847.2 ± 40.8^A.b^	13,648 ± 731^A.b^	109.31 ± 4.93^A.a^	103.41 ± 125.68^A.a^
	7	847.1 ± 3.1^A.b^	14,006 ± 379^AB.b^	106.83 ± 1.30^A.a^	82.63 ± 0.91^A.a^
	14	948.0 ± 12.2^AB.ab^	15,406 ± 389^A.ab^	110.15 ± 2.40^A.a^	119.04 ± 9.22^A.a^
	28	1027.8 ± 58.6^A.a^	16,529 ± 339^A.a^	105.27 ± 1.94^A.a^	75.69 ± 104.38^A.a^
E	1	919.3 ± 27.3^A.a^	14,462 ± 598^A.a^	105.82 ± 2.54^A.a^	52.74 ± 15.53^A.a^
	7	895.3 ± 22.8^A.a^	14,432 ± 558^AB.a^	112.25 ± 4.92^A.a^	27.00 ± 34.60^A.a^
	14	899.6 ± 80.8^AB.a^	14,523 ± 677^AB.a^	107.52 ± 1.84^A.a^	48.78 ± 46.50^A.a^
	28	956.3 ± 3.6^A.a^	15,239 ± 44^ABC.a^	109.25 ± 4.43^A.a^	38.20 ± 51.20^A.a^
F	1	854.3 ± 29.8^A.a^	13,511 ± 439^A.a^	107.32 ± 1.52^A.a^	60.67 ± 43.11^A.a^
	7	964.8 ± 93.7^A.a^	13,871 ± 762^AB.a^	110.27 ± 0.84^A.a^	9.16 ± 9.95^A.a^
	14	883.0 ± 76.7^AB.a^	14,337 ± 645^AB.a^	108.31 ± 0.96^A.a^	46.36 ± 16.38^A.a^
	28	834.8 ± 22.8^bc.a^	13,889 ± 749^CD.a^	110.63 ± 10.01^A.a^	116.29 ± 41.49^A.a^

*Note:* Values are provided as mean ± SD. Significant differences (*p* ≤ 0.05) between yogurt samples were determined with the Tukey test. Comparisons between storage conditions are expressed in lower case; comparisons between samples are expressed in upper case.

^a^ Control: Commercial culture combination, A: 6K1‐3B7, B: 10K1‐3B7, C: 10K2‐3B7, D: 4K6‐3B7, E: 3K4‐3B7, F: 5K3‐3B7.

### 3.6 Rheological Properties of Yogurt Samples

**FIGURE 4:**
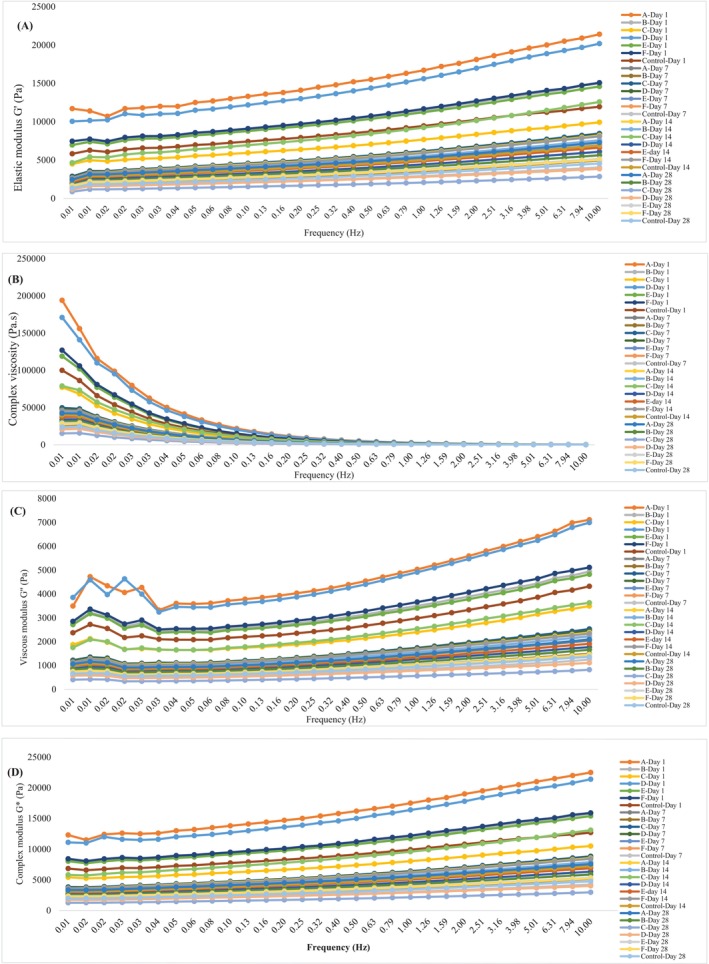
Yogurt samples: (A) Elastic modulus *G*′ (Pa) – frequency (Hz), (B) complex viscosity (Pa.s)—frequency (Hz), (C) viscous modulus *G*″ (Pa)—frequency (Hz), (D) rheology graphs against complex modulus G* (Pa)—frequency (Hz). Sample codes refer to A: 6K1‐3B7, B: 10K1‐3B7, C: 10K2‐3B7, D: 4K6‐3B7, E: 3K4‐3B7, F: 5K3‐3B7, Control: Commercial culture combination.

**FIGURE 5:**
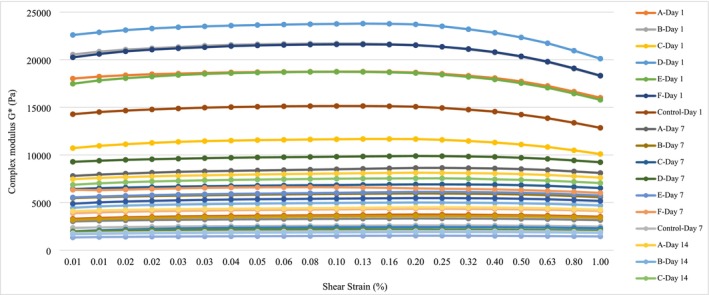
Strain (amplitude) screening test results of yogurt samples. Sample codes refer to A: 6K1‐3B7, B: 10K1‐3B7, C: 10K2‐3B7, D: 4K6‐3B7, E: 3K4‐3B7, F: 5K3‐3B7, Control: Commercial culture combination.

Frequency scanning test results of yogurt samples are shown in Figure 4. The samples were examined on four different parameters expressed as elastic modulus *G*′ (Pa), viscous modulus *G*″ (Pa), complex viscosity μ* (Pa.s), and complex modulus G* (Pa). The higher the elastic modulus parameter, the more viscous and less fluid the yogurt samples. Accordingly, considering the *G*′ parameter, samples A and B presented the highest values at the end of the shelf‐life (Figure 4A). There is an increase in *G*′ and *G*″ values of yogurt samples throughout their storage. Samples A and D had the highest values regarding all parameters on the 1st day of storage. At the beginning of storage, the highest *G*″ values were obtained in yogurt samples A and D, while the lowest *G*″ values were determined in control and C yogurt (Figure 4C). On the 7th and 14th days, C yogurt showed the highest *G*″ value, and on the 28th day it again showed the lowest *G*″ value. Strain scanning analysis results of yogurt samples are given in Figure 5. Analysis conditions were determined as 0.01%–1.0% at 5°C and strain scanning range at 1 Hz. On the 1st, 7th, and 28th days of the storage, the highest gel frequency was found in sample D, followed by samples F, A, and B.

### 3.7 Microstructure Analysis in Yogurt Samples

**FIGURE 6:**
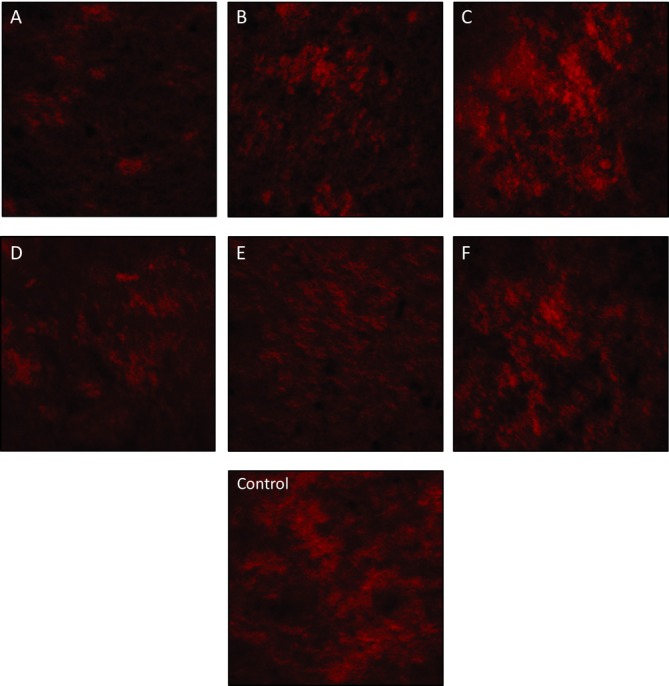
Confocal analysis results of yogurt samples. Sample codes refer to (A) 6K1‐3B7, (B) 10K1‐3B7, (C) 10K2‐3B7, (D) 4K6‐3B7, (E) 3K4‐3B7, (F) 5K3‐3B7, control: commercial culture combination.

Microstructure images of yogurt samples were taken with a laser scanning confocal microscope on the 28th day of storage. Confocal analysis images of yogurt samples are given in Figure 6. Although the WHC of F yogurt was higher than that of the other samples, considering its firmness value of the 28th day texture analysis, it was at a lower value than that of the other samples, which supports the image we examined in the confocal analysis. In general, no serious negative effects were observed in the microstructural structure of yogurts produced with novel cultures in terms of protein tightness or serum holes compared to control yogurts. Structure images did not contradict the results of syneresis, WHC, and texture analyses; on the contrary, they supported those results.

### 3.8 Sensory Properties of Yogurt Samples

**FIGURE 7:**
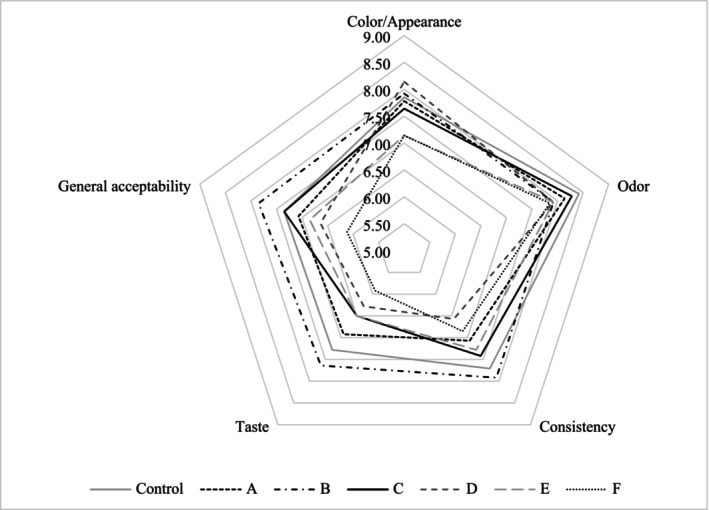
Sensory results of yogurt samples. Sample codes refer to A: 6K1‐3B7, B: 10K1‐3B7, C: 10K2‐3B7, D: 4K6‐3B7, E: 3K4‐3B7, F: 5K3‐3B7, control: commercial culture combination.

Sensory analyses of yogurt samples were assessed using a 9‐point scale (1—very bad, 9—very good) including color‐appearance, smell, consistency, taste, and general acceptability (GA) parameters. The sensorial scores of samples are presented in Figure 7. While evaluating the color‐appearance parameter, the smoothness of the structure of the yogurts taken from the samples with a spoon was taken into consideration. The highest score in terms of color‐appearance was determined in sample D, followed by B and control samples. In terms of the odor parameter, the highest value belonged to the control sample followed by C, A, and B samples. According to taste, consistency, and GA criteria, B yogurt received the highest score followed by control yogurt. On the contrary, the lowest taste, consistency, and GA scores were found in F and D yogurt.

## 4 Discussion

There is a statistical difference between the storages of yogurt samples B, C, E, and F during the shelf life, that is, on the 7th, 14th, and 28th days (*p* ≤ 0.05). However, the difference between the samples was detected only in yogurt samples A, C, D, E, and F on the last day of storage. The pH values varied between 3.90 and 4.51 during the storage period. It has been suggested that a pH value below 4 negatively affects bacterial viability and sensory acceptability (Ranadheera et al. 2012). As yogurt starter cultures continue to grow during storage, pH values decrease while TA values increase, due to the conversion of lactose into lactic acid and the hydrolysis of fats, leading to the formation of free fatty acids. Prasanna et al. (2013) demonstrated in their study on low‐fat yogurts produced with EPS‐producing bacteria that as storage time increased, pH levels decreased while TA increased. The increment in acidity value that occurs during storage has also been detected by other researchers (Güven et al. 2005; Mani‐López et al. 2014; Aktaş et al. 2022) in parallel with our findings.

WHC, one of the desired properties for yogurt quality, can be expressed as the water retention ability of the proteins in the yogurt curd and is an important structural feature of yogurt. Yogurt curds, which have high water retention properties, provide a tighter‐harder texture. Due to the acidification (pH falling to the isoelectric point) during fermentation, caseins tend to form a gel to trap serum by hydrogen bonding (Zhou et al. 2012). The reason for the statistically significant difference in WHCs of some samples during the storage period can be attributed to the rearrangement of protein network structures over time to obtain a tighter gel (Sözeri Atik et al. 2023). It is generally expected that the increased acidity level stimulates serum loss from the casein gel, leading to a decrease in its WHC. However, contrary to expectations, while pH values decreased during storage, WHC did not decrease for C and F yogurt samples. The reason for this may be the technological properties of the bacterial strains isolated from village‐type yogurts. Indeed, some of the properties expected in yogurts produced with commercial yogurt starter cultures cannot be obtained in yogurts produced with newly isolated wild cultures (Demirci et al. 2022).

Syneresis, or serum separation, that occurs in yogurts is an important technological defect resulting from extreme irregularity in clot stability, causing liquid accumulation on the gel surface and an undesirable sensory mouthfeel (El Bouchikhi et al. 2019). A direct relationship between gel firmness and syneresis has been shown (Arab et al. 2022). When the texture analysis results were examined, it was seen that the syneresis value was also low in yogurts with high firmness values at the end of the storage period. Numerous studies in the literature have reported a decrease in syneresis in yogurts during storage, which is consistent with our findings (Prasanna et al. 2013; Marchiani et al. 2016). Likewise, Gölbaşι et al. (2023) found that as the acidity increased in these yogurts, the syneresis decreased in line with our results in their study on village‐type homemade yogurts. In fact, one of the biggest defects in yogurt, syneresis, was lower in yogurt produced with novel wild cultures compared to yogurt produced with commercial cultures until the 14th day, which is a promising result.

Many commercial yogurt samples available on the market have a dry matter content of 14%–15% (Tolu and Altun 2021), which is in line with the findings of our study. At the same time, as stated in the TFC Fermented Dairy Products Communiqué, the fat content of yogurts produced from full‐fat milk should be at least 3.8% and the milk protein by mass should be at least 3% (Communiqué 2022). Regarding this, the fat and protein amounts of the yogurts produced in this study are among the values stated in the communiqué. According to the TFC Fermented Dairy Products Communiqué (communiqué no. 2022/44), it is stated that there must be at least 7 log CFU/g specific microorganisms in yogurt. The *L. bulgaricus* numbers determined in this study were among the values stated in the notification. At the same time, when comparison was made between storage days, the difference between all samples was found to be insignificant (*p* > 0.05). In parallel with our findings, studies have observed that there is a significant decrease in the number of *L. bulgaricus* gradually throughout the 28 days (Paseephol and Sherkat 2009; Öztürk et al. 2017). In this case, it is thought that the over‐acidification during shelf‐life causes the number of *L. bulgaricus* to decrease (Donkor et al. 2006). However, contrary to the results of our study, Sözeri Atik et al. (2023) reported that the number of *L. bulgaricus* in yogurts produced with newly isolated cultures from village yogurts showed a regular increase until the 14th day and then decreased, while in control yogurt there was an increase throughout the 28 days. In fact, in the early stages of yogurt storage, an increase in acid‐resistant *L. bulgaricus* strains can be seen with the increase in acidity. Then, after a while, the number of *L. bulgaricus* that cannot tolerate the acidity. However, this may not always be possible since it depends on factors such as the acidity tolerance of the *L. bulgaricus* used and its relationship with *S. thermophilus* .

Our results on the number of streptococci are consistent with the results of Sözeri Atik et al. (2023). In their studies with different wild yogurt cultures, the researchers found that the number of *S. thermophilus* decreased from the beginning to the end of storage in two yogurts, while the number of streptococci at the end of storage remained higher than that at the beginning of storage in the yogurt they produced with another combination. Celik et al. (2023) investigated microbial changes in yogurts produced with commercially available starter cultures and reported an increase in streptococci counts during storage. On the other hand, it should also be noted that the number of streptococci remained higher than the number of lactobacilli throughout storage. The higher number of streptococci compared to lactobacilli during yogurt storage has been reported by various researchers before (Gölbaşι et al. 2023; Sözeri Atik et al. 2023). In fact, with the current trend in the dairy industry towards milder yogurts with a good mouthfeel, yogurt culture providers and manufacturers prefer to keep the *S. thermophilus* count higher than the lactobacilli count (Sieuwerts 2016).

Textural and rheological properties, especially of set‐type yogurts, are of critical importance in terms of product quality, shelf‐life, and acceptability by consumers. These properties are especially affected by the composition of the milk, the total solid content, the heat treatment conditions, the type and ratio of starter culture, the fermentation process, and storage time (Wang et al. 2018; Sözeri Atik et al. 2023). Therefore, to determine the effect of different novel starter cultures used in this study on the textural properties of yogurt, the firmness, consistency, cohesiveness and viscosity properties of yogurts were examined throughout the storage period.

Yogurt firmness may vary depending on the starter cultures. The observed increases in the firmness values of all samples except for F yogurt samples at the end of the storage period may be due to EPS production. EPSs can be divided into two groups: homopolysaccharides and heteropolysaccharides. Both forms of EPS show high water retention ability (Mudgil et al. 2017; Arab et al. 2022). Considering that LAB produce EPS as a self‐protection mechanism under stress conditions (biotic, abiotic, competition, pH, temperature, etc.), it is not wrong to interpret that EPS production intensifies towards the end of storage against increasing acidity during storage and competition between starters that may occur in some cases (Jurášková et al. 2022). It is already known that yogurt starters, especially *S. thermophilus* , are strong EPS‐producing bacteria. Regarding the lower firmness values and higher syneresis values of yogurt produced with commercial cultures during storage, Han et al. (2016) used 19 of the high EPS‐producing *S. thermophilus* strains isolated from Chinese fermented products in yogurt production and observed much better texture and lower syneresis in these yogurts compared to commercial ones.

On the other hand, the decrease in syneresis values in yogurts during storage coincides with the increase in firmness values in our study. Consistent with our results, Sözeri Atik et al. (2023) also found much higher firmness values in 3 of the 4 yogurts produced by combining cultures isolated from local yogurts compared to those produced with commercial cultures. Although there were some minor fluctuations during the storage period, in general, consistency values increased at the end of storage compared to the beginning for all yogurts. In accordance with the results of Sözeri Atik et al. (2023), the highest consistency values were also obtained in yogurts with the highest firmness values. Mélo et al. (2024) also reported that the consistency figures in yogurts increased with storage days. On the other hand, in a similar study, Han et al. (2016) compared *L. bulgaricus* and *S. thermophilus* isolated from artisanal Chinese fermented dairy products with commercial cultures and observed the highest cohesiveness value in yogurt produced using novel cultures and the lowest values in yogurt manufactured with commercial ones. However, the *S. thermophilus* used by these researchers in yogurt production was the culture with the highest EPS production among 19 different *S. thermophilus* isolates. In summary, textural parameters were generally consistent with each other and with the syneresis results throughout the storage. It is a desirable result that the yogurt produced with the novel strains showed better firmness and consistency values compared to the yogurt produced with commercial cultures and that this situation did not show a negative fluctuation during storage.

μ* is a parameter obtained by frequency sweep tests and used to define rheological properties. As the μ* of a semi‐solid food increases, it indicates that it has a more elastic structure and solid‐like properties (Joyner 2019). According to the findings in Figure 4B, it was observed that the μ* decreased with increasing frequency and this confirmed the non‐Newtonian shear thinning behavior of the set‐type yogurt. These results are consistent with previous research that demonstrated similar behavior in set‐type yogurts (Khubber et al. 2021). Viscous modulus (*G*″) is a parameter that describes the viscosity properties of a material. Viscoelastic refers to the fluidity and viscosity behavior of a material. As the *G*″ value increases the water retention capacity increases. Indeed, the lowest syneresis values at the end of storage were found in C yogurt. The trend of the complex modulus (G*) with increasing frequency was a linear increase as shown in Figure 4D. G* refers to the network resistance of the gel or polymer against applied stress (Gabriele et al. 2001). In this study, the highest G* value belonged to sample A at the beginning of the storage. At the end of the storage, considering the G* values of the control group, samples A, B, E and F show the higher values while yogurts D and C have the lower ones. These results can be interpreted as the protein network of yogurt samples strengthened during shelf‐life. Although linear viscoelasticity was observed in all samples, significant differences were detected in G* values. This shows that the same protein interaction forces exist at different levels. Although the process conditions were kept constant, the main reasons for the G* differences were the occurrence of protein flocculation during cold storage and the difference in the restructuring kinetics of protein bands. As in the frequency scanning test, in the amplitude scanning test, yogurts produced with novel culture show higher gel firmness than the control group during the storage period. The samples that give the best results at the end of storage are F, D, and A yogurt samples.

Although frequency and stress sweep tests are widely used to evaluate the linear viscoelastic behavior of semisolid dairy products such as yogurt (Sözeri Atik et al. 2024), incorporating creep‐recovery tests can provide additional insight into their time‐dependent mechanical response (Oseli et al. 2024). Creep‐recovery analysis involves subjecting the material to constant shear stress for a duration and then eliminating it to determine structural integrity, long‐term deformation, and recoverability (Tan and Joyner 2019). Therefore, creep‐recovery analysis can be performed in subsequent studies to understand the stability of yogurts under mechanical stresses during storage. Consequently, the findings from the textural and rheological analyses align with each other. At the same time, it was observed that yogurt made with novel cultures from village‐type homemade yogurts gave better results than the control yogurt in many parameters, such as the rheological, textural, and WHC properties. It has been reported that the type and amount of polysaccharides produced by yogurt starter cultures significantly change the viscosity of yogurt, while rheological parameters also change in yogurt produced using different starters (Prete et al. 2021). Especially, the polysaccharide types produced by starters were found to have a strong relationship with yogurt rheology (Sözeri Atik et al. 2024). Similarly, there is a study showing that the use of EPS‐producing strains and non‐EPS‐producing strains together in a starter mix improves the rheological properties of yogurt (Prasanna et al. 2013). From this point of view, as shown in our study, the observation of better rheological properties in yogurt, especially in A and F yogurt compared to control yogurt, can be attributed to the fact that the type and amount of polysaccharide produced by these novel cultures are more suitable and more than those produced by the bacteria in the starter culture mix.

The results of microstructure analysis are important for texture defect and serum separation. The serum should be distributed homogeneously in the protein matrix and black areas should be at a minimum. An excessive serum area is an important indicator that the syneresis of the yogurt samples is high. Analysis of the images revealed that the casein micelle structure in yogurt sample A was looser compared to the other samples. Researchers have stated that there is a correlation between the microstructure of yogurt, its firmness, and its tendency to separate serum (Gilbert et al. 2020). In the light of this information, syneresis and WHC results also support this microstructural image.

Regarding the sensory analyses of yogurt, it is not surprising that yogurt B had the highest sensory scores in terms of syneresis values and texture parameters; at the same time, although statistically insignificant, yogurt B was found to be the yogurt with the highest fat value and ash value among all yogurts. The findings that the novel *S. thermophilus* strain 10K1, used to produce yogurt B, received higher scores in certain sensory parameters compared to commercial yogurt, while some other novel *S. thermophilus* strains were rated less favorably than the control yogurt, align with the results of a similar study conducted by Han et al. (2016).

According to the analysis results, there is a decrease in pH values and an increase in TA values in yogurt samples as bacterial growth continues during the storage period. During the storage period, the increasing acidity promoted serum loss from the casein gel, leading to a reduction in WHC. Contrary to expectations, while pH values decreased during storage, WHC did not decrease. Syneresis and WHC results in produced yogurts were compatible with each other. According to the microbial enumeration, streptococci and lactobacilli counts remained above 6.00 log CFU/g in all samples throughout storage. Considering the texture analysis, the weakest yogurt sample in terms of firmness was the control sample throughout the complete storage. The samples with the lowest consistency values were the control, F, and D, which also exhibited low firmness. Based on the rheological results, the A and B yogurt samples showed the highest values in the frequency sweep test at the end of storage, whereas the F and D yogurt samples exhibited the highest values in the strain (amplitude) sweep test. Yogurt samples made with cultures newly isolated from village‐type homemade yogurts, especially B, C, and D, gave better results than the control yogurt in many parameters, such as the rheological, textural, and WHC properties. Regarding the sensory analyses, no obstacles were identified for the commercialization of *S. thermophilus* strains 10K1, 10K2, and 4K6 for yogurt production. As a result, the quality parameters of yogurt produced with newly isolated cultures are not inferior to commercial yogurt. In this study, the researchers wanted to compare the bacteria isolated from yogurt produced in mountainous regions where commercial cultures were not used, with commercial cultures and obtained promising results. It is hoped to carry out further studies on the commercialisation of these new cultures and to provide recommendations to companies on suitable mixes.

## Author Contributions

**Begüm Denktaş:** data curation (equal), formal analysis (equal), investigation (equal), methodology (equal), visualization (equal), writing – original draft (equal). **Meryem Kübra Satılmış:** formal analysis (equal), investigation (equal), methodology (equal). **Hale İnci Öztürk:** formal analysis, Investigation, Methodology, Visualization, Writing – original draft, Writing – review and editing. **Talha Demirci:** conceptualization, Formal analysis, Investigation, Methodology, Validation, Writing – review and editing. **Nihat Akın:** conceptualization, Funding acquisition, Resources, Validation, Writing – review and editing.

## Conflicts of Interest

The authors declare no conflicts of interest.

## Data Availability

Data will be made available on request.
